# Adverse experiences resulting in emergency medical treatment seeking following the use of lysergic acid diethylamide (LSD)

**DOI:** 10.1177/02698811221099650

**Published:** 2022-06-07

**Authors:** Emma I Kopra, Jason A Ferris, James J Rucker, Benjamin McClure, Allan H Young, Caroline S Copeland, Adam R Winstock

**Affiliations:** 1 Department of Psychological Medicine, Institute of Psychiatry, Psychology & Neuroscience, King’s College London, London, UK; 2 Centre for Health Services Research, Faculty of Medicine, The University of Queensland, Brisbane, QLD, Australia; 3 South London and Maudsley NHS Foundation Trust, London, UK; 4 Belfast Health and Social Care Trust, Belfast, UK; 5 Institute of Pharmaceutical Science, Faculty of Life Sciences and Medicine, King’s College London, London, UK; 6 National Programme of Substance Abuse Deaths, Population Health Research Institute, St George’s, University of London, London, UK; 7 Institute of Epidemiology and Health Care, University College London, London, UK; 8 Global Drug Survey, London, UK

**Keywords:** Adverse effects, LSD, psychedelics, safety

## Abstract

**Background:**

Recreational lysergic acid diethylamide (LSD) use is growing in popularity amid increasing research interest on psychedelics and their possible therapeutic potential yet; the potent psychotropic effects of LSD may result in adverse reactions and behaviour.

**Aims:**

This study aimed to investigate the 12-month incidence and nature of LSD-related adverse experiences resulting in emergency medical treatment (EMT) seeking in an international sample of people reporting LSD use.

**Methods:**

We use data from the 2017 Global Drug Survey – a large anonymous online survey on patterns of drug use conducted between November 2016 and January 2017.

**Results:**

Out of 10,293 past-year LSD users, 102 (1.0%) reported seeking EMT, with a per-event risk estimate of 0.2%. Younger age, comorbid mental health conditions and higher frequency of use were associated with increased risk of EMT seeking. The most common symptoms were psychological, including anxiety, panic and confusion, with the most common explanatory factors cited by respondents being poor ‘setting’ and ‘mindset’. Most responders reported feeling back to normal within 24 h, but 11 participants experienced persistent issues after 4 weeks.

**Conclusion:**

The results suggest that LSD is a relatively safe drug in recreational settings. Adverse reactions are typically short-lived, self-limiting and psychological in nature. Sub-optimal set and setting were commonly reported as suspected contributory factors. Within clinical settings, patient screening, preparatory sessions and supervision should reduce these acute risks considerably.

## Introduction

Lysergic acid diethylamide (LSD) is a potent psychedelic drug. Like all classical hallucinogens, its main mechanism of action is partial agonism at central type 2A serotonin receptors, which is thought to mediate its psychedelic effects including euphoria, perceptual alterations (e.g. synaesthesia), enhanced introspection, feelings of transcendence, spiritual awareness and changes in sense of self, time and space (Liechti, 2017; Nichols, 2016). The common recreational dose of LSD ranges between 50 and 400 μg (Dolder et al., 2016), with psychedelic doses typically considered above 100 µg.

The most common adverse reactions associated with the use of LSD are psychological in nature and include anxiety, paranoia, loss of thought control, panic attacks and self-harming behaviour (Carhart-Harris et al., 2016; Martin, 1970; Schmid et al., 2015). Although most symptoms resolve spontaneously after the drug effects wear off, about 2% of people who use LSD report the drug have had negative effect on their well-being (Carhart-Harris and Nutt, 2010). Flashbacks, recurrences of perceptual alterations or other sensations experienced during the trip are reported by 10–35% of people who use the drug (Baggott et al., 2011; Carhart-Harris and Nutt, 2010; McGlothlin and Arnold, 1971), but are rarely perceived as unpleasant or harmful (Carhart-Harris and Nutt, 2010). Preliminary evidence suggests risk of serious acute reactions and flashbacks might be increased by presence of personal or familial mental health problems (Cohen, 1960; Strassman, 1984).

LSD has low toxicity relative to other psychoactive drugs and in normal doses induces only minor physiological effects including slight increases in heart rate and blood pressure (Dolder et al., 2017; Schmid et al., 2015). Only two known cases exist where massive LSD overdose appears to have been directly responsible for death (Nichols and Grob, 2018). Based on these case reports and evidence from animal studies, the lethal dose of LSD has been estimated as roughly a thousand times or more the usual recreational dose (Nichols and Grob, 2018). Other deaths initially attributed to LSD toxicity have later been attributed to prone maximal restraint by police and/or the use of other psychoactive substances (Nichols and Grob, 2018). Since the conception of National Programme on Substance Abuse Deaths (NPSAD) in 1997, which compiles drug-related death case reports from coroners on a voluntary basis from England, Wales and Northern Ireland, LSD use has been determined as directly implicated in causing death in only two cases; one being a suicide following the combined use of LSD and cannabis, and one either a jump or a fall from a 10th-floor window (Copeland, 2021).

It is now widely recognized that contextual factors and person characteristics and mindset are crucial in determining the nature of a psychedelic experience (Carhart-Harris et al., 2018). Modern experimental studies using psychedelics have reported largely positive outcomes and no serious adverse reactions (Fuentes et al., 2020), which might be attributed to close attention being paid to dosing regimens, inclusion criteria and ‘set’ and ‘setting’. In contrast, a proportion of studies between the ‘50s and ‘70s neglected these factors or even manipulated the environment in a negative way, subsequently observing worse outcomes (Albarelli, 2009; Carhart-Harris et al., 2018; Oram, 2014). Misinformation about the dangers of LSD circulated via mass media during the ‘60s is believed to have contributed to more ‘bad trips’ during this era also in the community, through negatively affecting the mindset and expectations of people about to use the drug (Bunce, 1979). Psychedelics are nowadays generally portrayed in a more positive light in the media, but concerns have been raised that overly positive media reports could understate risks (Carhart-Harris et al., 2018; Yaden et al., 2020). National surveys have shown trends of increasing LSD use over the past decade (gov.uk, 2010, 2019; Yockey et al., 2020), and although majority seem more educated about the safe use of LSD than previously, partially due to more available and accurate harm reduction information (see, for example, GDS highway code; Global Drug Survey (GDS), 2014), adverse reactions still occur. Neglect of important safety precautions could at worst lead to another ‘negative cultural feedback loop’; in essence, resulting negative psychedelic experiences contribute to negative public opinion about psychedelics and vice versa (Carhart-Harris et al., 2018).

Information about the nature and predictors of LSD-related adverse reactions is important both for prevention and for advising public and medical professionals about acute management when incidents do occur. The modest amount of previous literature on serious adverse psychedelic experiences to date have been by large part derived from official records, often limited by uncertainty regarding the exact circumstances surrounding incidents, quantity and quality of substances used and the presence of potential biases and inaccuracies in reporting. While self-report surveys also hold some of these limitations, they can help supplement data from official records by providing more detailed, firsthand insights on the nature, reasons and consequences of individuals’ experiences (Coney et al., 2017). This study is an exploratory analysis of the occurrence, predictors and nature of adverse experiences resulting in emergency medical treatment (EMT) seeking following LSD use, in a large international sample of GDS respondents. Specifically, we investigate the potential of demographic variables, mental health conditions, use patterns and previous LSD experience as predictors of EMT incidents and explore the symptom profile and recovery time from these experiences, concomitant use of other substances, perceived reasons for incidents and experiences’ impact on subsequent substance use.

## Methods

### Design

This investigation is one part of two papers looking at EMT seeking in response to psilocybin mushroom and LSD use in the same survey (Kopra et al., 2022). The reported methods are substantially similar within the two papers but are reproduced in each for the convenience of the reader.

The GDS is an annual, anonymous and encrypted online survey on substance use. It is advertised in social networking sites in collaboration with media partners and harm reduction organizations. Using a self-nominating sampling method, the survey can effectively reach large amounts of respondents engaging in rarer practices and stigmatized behaviours, who would be difficult to access through representative sampling frames. The survey includes of a core set of questions on basic patterns of drug use that remain the same each year, besides annually changing specialist sections on more specific topics.

GDS2017 was launched in November 2016 and was available until January 2017, in 10 languages. Participants were not remunerated. Full details about the survey design and recruitment, including related discussion on the survey’s utility, can be found elsewhere (Barratt et al., 2017). Multi-institutional ethics approval was obtained from the King’s College London Research Ethics Committee (11671/001: GDS), University of Queensland (No. 2017001452) and The University of New South Wales (HREC HC17769) Research Ethics Committees. Access to the relevant sections of the GDS2017 data set (demographic data and sections on psychedelics) was obtained through a data-sharing agreement with the GDS.

### Measures

At the start of the survey, a wide range of demographical information was collected. In subsequent sections, participants were asked to indicate when they last used specific drugs from an extensive list of substances including LSD (never, in the last 30 days, between 31 days and 12 months ago, more than 12 months ago). Those indicating history of use with a drug were then redirected to sections with in-depth questions about the use of these substances. Among other questions, people who reported past-year LSD use were asked about the number of days they used the drug in the last 12 months; whether they used LSD for the first time in the last 12 months; the number of doses they normally take on a day they use LSD; and whether they had sought EMT following the use of LSD in the past year. The number of EMT incidents experienced was not recorded.

Those indicating having sought EMT were directed to a further set of questions about this, a specialist section included in the 2017 survey. Respondents were asked to tick the psychological and physiological symptoms they presented with from a list of 20, extrapolated from the available literature. Respondents were also asked the number of LSD doses they had consumed during that session, what (if any) other substances they had taken, the duration of symptoms and whether they had required hospitalization. Participants were then asked about why they thought the incident occurred, picking a maximum of three out of six options, and enquired about the impact of their experience on their subsequent use of LSD and other substances.

Towards the end of the survey, all participants were asked about their overall well-being and mental health, including whether they have ever been diagnosed with a mental illness. Ethic review boards required that participants were allowed to skip questions and leave empty responses if they did not want to complete specific items.

### Data analysis

Per-event risk of seeking EMT was calculated by dividing the number of participants indicating past-year EMT seeking with the total number of times LSD was used among past-year users, specifically:

$$\frac{N\;\mathrm{participants}\;\mathrm{reporting}\;\mathrm{EMT}}{\left[\begin{array}{l} \mathrm{Mean}\;\mathrm{times}\;\mathrm{used}\;\mathrm{past}\;\mathrm{year}\times \\ \;\;\;\;\;\;\;\;\;\;\;\;\;\;N\;\mathrm{past}\;\mathrm{year}\;\mathrm{users} \end{array}\right]}$$

Only those participants responding to the EMT question were included when calculating the estimated total times used (the denominator), therefore creating a representative sample of those proceeding and choosing to respond to the EMT question. While median and interquartile range (IQR) of past-year LSD uses were used for descriptive data, mean was used in the above calculation for the most accurate estimate of total times used in the sample.

Non-parametric statistics were utilized because dependent variables were found to be non-normally distributed. Mann–Whitney *U* tests were used to investigate whether there were differences in the age, past-year frequency of use, or number of doses used per day of use between EMT seekers and non-seekers. Pearson’s chi-square (χ^2^) tests were used to investigate associations between treatment seeking status and gender (male/female), previous LSD experience status (first time in the past year/experienced) and presence of mental health diagnosis (yes/no). Descriptive statistics and graphs were created to explore the experiences and symptom profiles of EMT seekers. In addition, two multiple correspondence analyses (MCAs; see Supplementary Methods and Abdi and Valentin, 2007) were conducted to explore pattern of relationships between different self-reported symptoms and between different self-reported reasons for incidents.

For all statistical analyses, complete case analysis was used, that is, responses with missing data on the variables of interest were excluded from those analyses. Analyses were performed using SPSS IBM Statistics 26.

## Results

### Frequency and risk of EMT incidents

**Figure 1.**
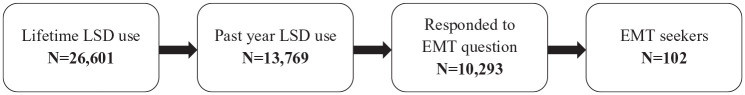
Question response flow chart.

A total of 119,108 respondents took part in the GDS2017, of which 22.3% (*n* = 26,601) reported lifetime use of LSD; 51.8% (*n* = 13,769) of those who reported lifetime LSD use reported using LSD within the past year; demographic profile of these participants is presented in Table 1. Of the 10,293 participants responding to the EMT question, 1.0% (*n* = 102) indicated they had sought EMT following LSD use in the past year (Figure 1).

**Table 1.** Demographic profile of past-year LSD users.

	*N*	Valid %^a^
*N* (% of lifetime users)	13,769	51.8^b^
*Age*		
<25	8802	63.9
25–34	3874	28.1
35+	1093	7.9
*Gender*		
Male	10,967	79.6
Female	2802	20.4
*Country of residence*		
Germany	2719	19.7
USA	2703	19.6
UK	1127	8.2
Denmark	1039	7.5
Canada	793	5.8
Australia	693	5.0
Other	4695	34.1
*Ethnicity*		
White	6959	84.8
Hispanic/Latino	533	6.5
Mixed	384	4.7
Other	332	4.0
*Mental health diagnosis*		
None	6148	73.8
Yes^c^	2187	26.2
Depression	1583	19.0
Anxiety	1120	13.4
ADHD	526	6.3
Bipolar	216	2.6
Psychosis	98	1.2
Other	396	4.8
*Use patterns*		
Past month users	4590	33.3
Past year novel users	4271	41.6
Median days past year use (IQR)	2	1–5

a Percentage when missing data excluded.

b Proportion of lifetime users.

c Those with a diagnosis were able to tick more than one diagnosis, hence the total number of these being larger than the number of respondents responding ‘Yes’.

Among responders to the EMT question, mean number of past-year LSD uses was 5.21 (SD = 11.60), resulting in the estimated 53,627 number of total times used. With 102 EMT seekers, this gave the per-event risk estimate of 0.001902, indicating 0.2% of past-year LSD uses led to EMT seeking in this sample.

### Predictors of EMT seeking

Comparing the characteristics of EMT seeking groups, Mann–Whitney *U* test revealed a significantly lower median age among EMT seekers (Mdn = 21, IQR = 18–25) compared to non-seekers (Mdn = 22, IQR = 19–27); Mann–Whitney test *z* = 2.96, *p* = 0.003. Chi-square analysis showed EMT seeking was significantly more prevalent among people with lifetime diagnoses of mental health conditions (1.8%) than those without (0.7%), χ^2^(1, *N* = 8299) = 19.33, *p* < 0.001. There was no difference in the prevalence of EMT seeking between men (1.0%) and women (0.9%), χ^2^(1, *N* = 10,293) = 0.12, *p* = 0.725.

Regarding patterns and history of use, no significant difference was found in the prevalence of EMT seeking between those who had used LSD for the first time in the past year (1.0%) compared to those with previous experience (1.0%), χ^2^(1, *N* = 10,235) = 0.04, *p* = 0.845. There was also no difference between the number of doses used per day of use between seekers (Mdn = 1.0, IQR = 1.0–2.0) and non-seekers (Mdn = 1.0, IQR = 1.0–2.0), Mann–Whitney test *z* = 0.914, *p* = 0.361. However, EMT seekers reported significantly higher past-year frequency of use (Mdn = 3.0, IQR = 2.0–8.5) compared to non-seekers (Mdn = 2.0, IQR = 1.0–5.0), Mann–Whitney test *z* = 2.18, *p* = 0.029.

### Symptom profile and nature of EMT incidents

Frequency of different reported symptoms is shown in Table 2. The median (IQR) number of reported symptoms was 5.0 (2.0–7.0). The five most commonly occurring symptoms were anxiety/panic (69.6%), confusion (64.7%), paranoia/suspiciousness (49.0%), seeing/hearing things (45.1%) and extreme agitation (39.2%). Observation of the MCA factor map (Supplementary Figure S1) indicated some division between psychological and physiological symptom presentations. Many psychological symptoms such as anxiety/panic and confusion tended to co-occur and, except from nausea, be unrelated or inversely related to physiological symptoms. Conversely, another cluster demonstrated close relationships between palpitations, chest pain and difficulty breathing as well as accident/trauma.

**Table 2.** Self-reported symptoms.

	*N*	%
Anxiety/panic	71	69.6
Confusion	66	64.7
Paranoia/suspiciousness	50	49.0
Seeing/hearing things	46	45.1
Extreme agitation	40	39.2
Memory loss	29	28.4
Extreme sweating	27	26.5
Palpitations	26	25.5
Very low mood in days afterwards	23	22.5
Thoughts or acts of self-harm	21	20.6
Difficulty breathing	21	20.6
Aggression/violence	18	17.6
Nausea/vomiting	18	17.6
Accident/trauma	17	16.7
Other	16	15.7
Chest pain	13	12.7
Fits/seizures	13	12.7
Passed out/unconscious	12	11.8
Headache	7	6.9
Bladder/kidney problems	4	3.9

**Figure 2.**
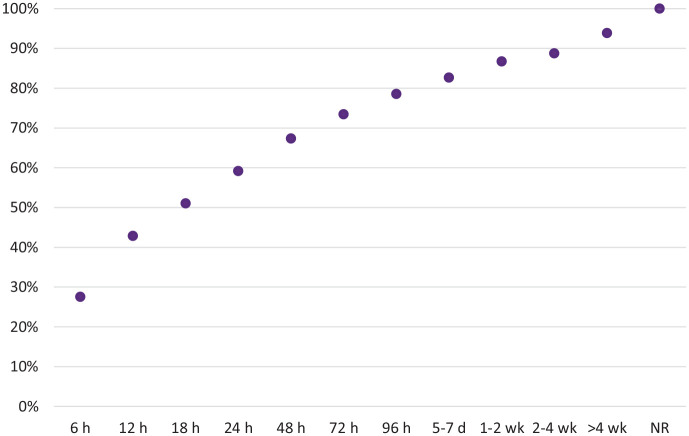
Time to recovery (NR = not recovered).

Slightly over half of EMT seekers (54.5%) reported being admitted to hospital. Figure 2 shows the length of time it took for participants to feel back to normal; majority of responders (59.2%) returned to normality within 24 h, but 11.2% experienced ongoing consequences beyond 4 weeks after LSD consumption.

The median number of LSD doses consumed was 2.0 (IQR = 1.0–3.0). Table 3 shows other substances participants had consumed in the lead-up to seeking EMT. In total, 50.0% of patients reported having used cannabis during the session, while alcohol consumption was reported by 23.5%.

**Table 3.** Other substances used preceding the incident.

	*N*	%
Cannabis	51	50.0
Alcohol	24	23.5
MDMA	12	11.8
Benzodiazepines	6	5.9
Cocaine	5	4.9
Amphetamine	4	3.9
Other	4	3.9
NPS	3	2.9
Ketamine	2	2.0
2C-B	1	1.0
Mephedrone	1	1.0
Opioids	1	1.0
Nothing else	33	32.4

MDMA = 3,4-Methyl enedioxy methamphetamine; NPS = New psychoactive substances.

**Figure 3.**
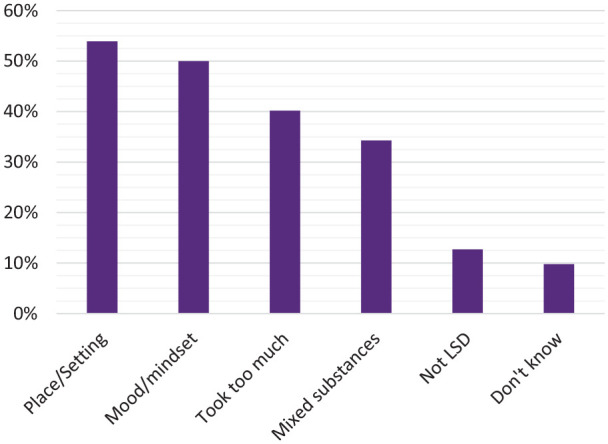
Self-reported reasons for the incident.

Reasons for why participants thought the incident had happened are presented in Figure 3. The most common reasons were wrong setting (53.9%) and wrong mindset (50.0%), while taking too much (40.2%) and mixing with other drugs (34.3%) were also frequently reported. Observation of the MCA factor map (Supplementary Figure S2) indicated that the two most reported reasons, wrong setting and wrong mindset, very commonly co-occurred.

As a result of their experience, 62.7% of EMT seekers reported having cut down their LSD use, while 16.7% reported no change in their LSD use; 35.3% reduced and 4.9% increased their other illicit drug or alcohol use.

## Discussion

This study examined the risk, predictors and nature of LSD-related adverse experiences leading to emergency medical presentations, using data from what to our knowledge is the world’s largest survey on substance use. Consistent with expectations and the previous literature, EMT incidents were relatively rare, occurring in 1.0% of people reporting past-year LSD use. This prevalence rate was similar to the ones observed in recent years’ GDS surveys (0.9%, 1.0% and 0.8% in GDS2019, 2020 and 2021, respectively; Winstock et al., 2019, 2021). Most commonly reported symptoms were psychological such as anxiety, panic and confusion, but physiological reactions as well as accidents and trauma also occurred. A large majority of respondents were able to identify reasons for their adverse experiences, suggesting these incidents may be preventable with public policy that focusses more on harm reduction and less on criminalization of end-users (Haden et al., 2016).

In most incidents, respondents returned back to normality within 24 h; however, 11 respondents experienced ongoing consequences beyond 4 weeks after LSD consumption. Although rare, examining the nature and predictors of any long-term effects would be of high priority to help provide support to such individuals and prevent similar incidents from occurring. Surprisingly, as many as 54.5% of EMT seekers reported being admitted to hospital. The exact meaning of admission to hospital was not defined in the survey and therefore this has to be interpreted with a caution.

The most common reasons reported for incidents were ‘wrong setting’ and ‘wrong mindset’. This is consistent with extensive evidence on the crucial role of ‘set and setting’ in determining the nature of psychedelic experiences – ‘set’ referring to individual’s pre-existing psychological factors, mood and expectations, and ‘setting’ to individual’s physiological and sociocultural environment (Carhart-Harris et al., 2018). While these aspects are arguably important for the safe use of any substance, psychedelics are thought to enhance sensitivity to context, which may magnify any negative internal and external influences (Carhart-Harris and Friston, 2019). Without adequate support, subsequent adverse reactions can escalate into, for example, panic attacks, paranoia, aggression, self-harming or accidents, as demonstrated in both the present survey and previous literature (Barrett et al., 2016; Carbonaro et al., 2016; Strassman, 1984).

We found younger age to be associated with higher frequency of EMT seeking. The association could be mediated by relatively less experience with LSD; however, separate analysis on the effect of previous experience on EMT outcomes was not significant. Younger age has previously been found to be related with EMT seeking following the use of synthetic cannabinoids (Winstock and Barratt, 2013) as well as with riskier injecting behaviours (Horyniak et al., 2013); lower risk-averseness and higher impulsivity of young people (Spear, 2000; Steinberg et al., 2008) could link to more spontaneous drug use in less ideal set and settings, and further increase risky behaviours when intoxicated, including substance mixing. Relative difficulty of emotional regulation in some younger people (Carstensen et al., 2011; Williams et al., 2006) might further limit the ability to navigate psychologically challenging experiences and LSD-induced increases in emotional lability (Carhart-Harris et al., 2016). Our findings are in line with previous studies on another psychedelic psilocybin, showing a relationship between younger age and higher difficulty of challenging experiences, impaired control and cognition and less blissful state during drug effects (Carbonaro et al., 2016; Studerus et al., 2012). Of note, young age has also been associated with higher degree of psychedelic-induced mystical-type experiences, suggesting younger people might overall be more sensitive to psychedelics’ effects, including positive ones (Russ et al., 2019). Regardless, we highlight that although significant, the sizes of associations between age and drug effects or outcomes were small in both the cited studies and the present survey.

Presence of mental health conditions were, likewise, associated with EMT incidents. While there is some promise of psychedelic-assisted treatments for people with some forms of mental health conditions (Fuentes et al., 2020; Rucker et al., 2016), these findings might help to highlight the importance of the interplay between pharmacological and extra-pharmacological factors in determining the direction of outcome (Brouwer and Carhart-Harris, 2020). In essence, psychedelics may represent a certain form of psychological ‘gamble’ for people already at risk of vulnerable, unstable mental states, but the dice are loaded not just by the state of mind of the individual, but also by their surroundings (Johnson et al., 2008; McCabe, 1977). Considering increasing reports of people aiming to self-treat their conditions with psychedelics in non-therapeutic contexts, our findings have important implications for harm reduction messaging and underline the importance of psychedelic preparation, support and integration (Gorman et al., 2021). The sample size did not allow for comparison of outcomes between different mental health conditions, investigation of which would require millions of respondents using LSD given the rarity of EMT seeking as well as of certain mental health conditions.

Among those medicated for their mental health conditions, drug–drug interactions are another plausible contributory factor to adverse experiences. Recent results from a clinical trial with psilocybin indicated that at least in normal doses, classical psychedelics can be safely administered to patients currently medicated with selective serotonin reuptake inhibitors (SSRIs; COMPASS Pathways, 2021), but the risk of serotonin toxicity remains when serotonergic drugs are used in higher doses or in combination with a monoamine oxidase inhibitor (MAOI; Malcolm and Thomas, 2021). In addition, the mood-stabilizer lithium has recently emerged as a drug of major concern when used together with psychedelics, with reports of severe adverse reactions including seizures and fugue states (Nayak et al., 2021).

‘Taking too much’ was a commonly reported reason for incidents. Unsurprisingly, doses taken on sessions leading to EMT were higher than usual. In half of the cases, subjects had also consumed cannabis in the lead-up to the incident. Cannabis can be used to enhance the psychedelic experience but may likewise trigger unpleasant psychotic-like symptoms also prevalent in the present survey (D’Souza et al., 2004), and it is possible that the combination of cannabis and LSD may be more risky in this regard than either alone. Furthermore, alcohol was consumed by nearly a quarter. The causal role of these substances in exacerbating adverse reactions is unclear as, for instance, some respondents might have attempted to alleviate anxiety or other unpleasant states by using these substances, and especially heavy alcohol use may correlate with suboptimal settings such as nights out. Regardless, over a third of participants reported ‘mixing drugs’ as a reason for incidents, suggesting the substances appeared to have played a role in a large proportion of cases of EMT seeking.

This study has some limitations. Self-nominating, non-probability sampling does not fulfil the criteria of traditional epidemiological research for public health, being subject to sampling and volunteer biases compromising the sample representativeness. In essence, people who are reached by the recruitment strategy and who choose to volunteer to participate might be inherently different to those who do not, due to, for instance, having specific interests in the topics of the survey (Eysenbach and Wyatt, 2002). Regardless, GDS has face validity for examining patterns of drug use within drug-using communities because it is comprehensive, anonymous and unconnected with government or law enforcement agencies (Barratt et al., 2017). Investigation of especially rare events, such as that investigated in this study, would require a very costly study for representativeness in community (King and Zeng, 2001). With purposive sampling targeting people who use substances, we were able to detect enough cases to obtain reasonable confidence on the occurrence of incidents as well as enough power to establish predictors, in a feasible and cost-effective manner.

There was, nevertheless, a lack of diversity in our sample, with predominantly young, male participants of white ethnicity, limiting the generalizability of results outside these populations. Although young white males partially reflect the common demographic profile of people who use LSD (Yockey et al., 2020), it is also possible that the reach of the GDS survey also favours these demographics (Barratt et al., 2017). Increasing survey translations to non-European languages and advertising in a broader range of media outlets targeting underrepresented demographics could help address these issues in future surveys. Increased representation of females, those of other gender, and ethnic minorities is particularly important given these demographics are highly affected by the mental health conditions psychedelic treatments are expected to be used for in the future (Becerra-Culqui et al., 2018; Williams, 2018; World Health Organization, 2017).

GDS relies on retrospective self-reports, hence recall and response biases are a concern. Specifically, answers might be affected by substances’ effects on perception and memory, which might be particularly pronounced with the types of serious adverse reactions examined in this study. Answers could also be biased by personal opinions about drugs and conscious attempts to influence survey results, through either downplaying or exaggerating adverse reactions. Although otherwise these problems are equally present also in representative population surveys, there is evidence psychedelic surveys might disproportionately reach and attract people holding positive attitudes about psychedelics (Haijen et al., 2018). However, given our survey is not specific to psychedelics but instead enquires about drug use in general, our sample may be less biased in this regard. In addition, the survey by Haijen et al. (2018) was prospective and only recruited people planning to take a psychedelic in the near future – therefore presumably less likely than our retrospective study to reach participants who, potentially due to their previous adverse experiences, hold more negative attitudes towards psychedelics and do not plan to take them again. It is also reassuring that neither baseline attitudes towards psychedelics nor the intensity of challenging psychedelic experiences predicted subsequent dropout rates of the same survey (Hübner et al., 2020). Theoretically this could apply to concerns regarding potential differences between those GDS respondents who choose to respond and finish all psychedelic-related sections and questions and those who do not.

Purity and strength of the substances cannot be confirmed from this survey, so it remains possible that a proportion of EMT incidents were due to drugs that were mistaken for LSD. *N*-(2-methoxybenzyl)phenethylamines (NBOMes) and other LSD analogues have been associated with fatalities and serious intoxications (Zawilska et al., 2020); these might have accounted for some incidents also in this survey, given only a minority of people who use LSD test their substances before use (Petranker et al., 2020). Furthermore, the variable ‘number of doses’ is vague, with potentially high variability within each possible answer. The survey also did not enquire about the potential occurrence of multiple EMT incidents per participant; given a number of these have likely occurred, the per-event risk of 0.2% is an underestimate as its calculation assumed only one EMT incident per participant.

Finally, given the data were collected from 2016 to 2017, it is not ruled out aspects of use or help-seeking behaviours influencing the prevalence, predictors or quality of reported adverse experiences have changed in a way or another over the past 5 years. The prevalence of LSD-related EMT incidents has remained fairly consistent over previous years’ GDS surveys (Winstock et al., 2019, 2021), although a drop from 1.0% to 0.8% was observed between GDS2020 (data from 2019, i.e. before the COVID-19 pandemic) and GDS2021. Reductions in the prevalence of EMT seeking were observed across most substances between these years (Winstock et al., 2021); possible explanations include safer contexts of use and/or reduced help seeking during the pandemic.

We are mindful that incidents in the GDS survey likely represent only a proportion of LSD-related adverse events, most likely those of more severe presentations. Even some serious reactions might not result in EMT due to, for instance, fear of legal consequences; in turn, a proportion of EMT visits might be ‘false alarms’, initiated, for example, by concerned family members of the individual despite a lack of symptoms, cases of which have been described previously (Leonard et al., 2018). Crucially, GDS did not survey in detail the exact circumstances surrounding the incidents, nor did we find out the determining factors leading to EMT seeking in each case. It is also unknown which or to what extent reported symptoms were induced by LSD versus other drugs consumed. Uncovering these details would require further investigations, potentially incorporating qualitative methods.

Regardless, this study provided a useful evaluation of LSD-related EMT incidents in the largest sample to date. The results suggest that LSD is a relatively safe drug in recreational settings. Adverse reactions are typically short-lived, self-limiting and psychological in nature. Sub-optimal set and setting were commonly reported as suspected contributory factors. Our findings can both help inform harm reduction efforts contributing to safer LSD use in the community as well as inform experimental research of potential risks.

## Supplemental Material

sj-docx-1-jop-10.1177_02698811221099650 – Supplemental material for Adverse experiences resulting in emergency medical treatment seeking following the use of lysergic acid diethylamide (LSD) Click here for additional data file. Supplemental material, sj-docx-1-jop-10.1177_02698811221099650 for Adverse experiences resulting in emergency medical treatment seeking following the use of lysergic acid diethylamide (LSD) by Emma I Kopra, Jason A Ferris, James J Rucker, Benjamin McClure, Allan H Young, Caroline S Copeland and Adam R Winstock in Journal of Psychopharmacology

## References

[bibr1-02698811221099650] Abdi H Valentin D (2007) Multiple correspondence analysis. Encyclopedia of Measurement and Statistics 2(4): 651–657.

[bibr2-02698811221099650] Albarelli HP (2009) A Terrible Mistake: The Murder of Frank Olson and the Cia’s Secret Cold War Experiments. Walterville, OR: Trine Day.

[bibr3-02698811221099650] Baggott MJ Coyle JR Erowid E , et al. (2011) Abnormal visual experiences in individuals with histories of hallucinogen use: A Web-based questionnaire. Drug and Alcohol Dependence 114(1): 61–67. 21035275 10.1016/j.drugalcdep.2010.09.006

[bibr4-02698811221099650] Barratt MJ Ferris JA Zahnow R , et al. (2017) Moving on from representativeness: Testing the utility of the Global Drug Survey. Substance Abuse: Research and Treatment 11: 1–17. 10.1177/1178221817716391 PMC5595253 28924351

[bibr5-02698811221099650] Barrett FS Bradstreet MP Leoutsakos JS , et al. (2016) The Challenging Experience Questionnaire: Characterization of challenging experiences with psilocybin mushrooms. Journal of Psychopharmacology (Oxford, England) 30(12): 1279–1295. 10.1177/0269881116678781 PMC5549781 27856683

[bibr6-02698811221099650] Becerra-Culqui TA Liu Y Nash R , et al. (2018) Mental health of transgender and gender nonconforming youth compared with their peers. Pediatrics 141(5): e20173845. 10.1542/peds.2017-3845 PMC5914494 29661941

[bibr7-02698811221099650] Brouwer A Carhart-Harris RL (2020) Pivotal mental states. Journal of Psychopharmacology 35: 319–352. 33174492 10.1177/0269881120959637 PMC8054165

[bibr8-02698811221099650] Bunce R (1979) Social and political sources of drug effects: The case of bad trips on psychedelics. Journal of Drug Issues 9(2): 213–233.

[bibr9-02698811221099650] Carbonaro TM Bradstreet MP Barrett FS , et al. (2016) Survey study of challenging experiences after ingesting psilocybin mushrooms: Acute and enduring positive and negative consequences. Journal of Psychopharmacology (Oxford, England) 30(12): 1268–1278. 10.1177/0269881116662634 PMC5551678 27578767

[bibr10-02698811221099650] Carhart-Harris RL Friston KJ (2019) REBUS and the anarchic brain: Toward a unified model of the brain action of psychedelics. Pharmacological Reviews 71(3): 316–344. 31221820 10.1124/pr.118.017160 PMC6588209

[bibr11-02698811221099650] Carhart-Harris RL Nutt DJ (2010) User perceptions of the benefits and harms of hallucinogenic drug use: A web-based questionnaire study. Journal of Substance Use 15(4): 283–300.

[bibr12-02698811221099650] Carhart-Harris RL Roseman L Haijen E , et al. (2018) Psychedelics and the essential importance of context. Journal of Psychopharmacology (Oxford, England) 32(7): 725–731. 10.1177/0269881118754710 29446697

[bibr13-02698811221099650] Carhart-Harris RL Kaelen M Bolstridge M , et al. (2016) The paradoxical psychological effects of lysergic acid diethylamide (LSD). Psychological Medicine 46(7): 1379–1390. 26847689 10.1017/S0033291715002901

[bibr14-02698811221099650] Carstensen LL Turan B Scheibe S , et al. (2011) Emotional experience improves with age: Evidence based on over 10 years of experience sampling. Psychology and Aging 26(1): 21–33. 20973600 10.1037/a0021285 PMC3332527

[bibr15-02698811221099650] Cohen S (1960) Lysergic acid diethylamide: Side effects and complications. The Journal of Nervous and Mental Disease 130: 30–40. 13811003 10.1097/00005053-196001000-00005

[bibr16-02698811221099650] COMPASS Pathways (2021) COMPASS Pathways Announces Positive Outcome of 25Mg COMP360 Psilocybin Therapy as Adjunct to SSRI Antidepressants in Open-label Treatment-resistant Depression Study. Available at: https://compasspathways.com/positive-outcome-25mg-comp360-psilocybin-therapy-adjunct-ssri-antidepressants-open-label-treatment-resistant-depression-study/

[bibr17-02698811221099650] Coney LD Maier LJ Ferris JA , et al. (2017) Genie in a blotter: A comparative study of LSD and LSD analogues’ effects and user profile. Human Psychopharmacology 32(3): 2599. 10.1002/hup.2599 28517366

[bibr18-02698811221099650] Copeland C (2021) National Programme on Substance Abuse Deaths (NPSAD). London: St George’s, University of London.

[bibr19-02698811221099650] Dolder PC Schmid Y Haschke M , et al. (2016) Pharmacokinetics and concentration-effect relationship of oral LSD in humans. International Journal of Neuropsychopharmacology 19(1): pyv072. 10.1093/ijnp/pyv072 PMC4772267 26108222

[bibr20-02698811221099650] Dolder PC Schmid Y Steuer AE , et al. (2017) Pharmacokinetics and pharmacodynamics of lysergic acid diethylamide in healthy subjects. Clinical Pharmacokinetics 56(10): 1219–1230. 28197931 10.1007/s40262-017-0513-9 PMC5591798

[bibr21-02698811221099650] D’Souza DC Perry E MacDougall L , et al. (2004) The psychotomimetic effects of intravenous delta-9-tetrahydrocannabinol in healthy individuals: Implications for psychosis. Neuropsychopharmacology: Official Publication of the American College of Neuropsychopharmacology 29(8): 1558–1572. 15173844 10.1038/sj.npp.1300496

[bibr22-02698811221099650] Eysenbach G Wyatt J (2002) Using the Internet for surveys and health research. Journal of Medical Internet Research 4(2): E13. 10.2196/jmir.4.2.e13 PMC1761932 12554560

[bibr23-02698811221099650] Fuentes JJ Fonseca F Elices M , et al. (2020) Therapeutic use of LSD in psychiatry: A systematic review of randomized-controlled clinical trials. Frontiers in Psychiatry 10: 943. 32038315 10.3389/fpsyt.2019.00943 PMC6985449

[bibr24-02698811221099650] Global Drug Survey (2014) GDS: The Highway Code. Available at: https://www.globaldrugsurvey.com/brand/the-highway-code/

[bibr25-02698811221099650] Gorman I Nielson EM Molinar A , et al. (2021) Psychedelic harm reduction and integration: A transtheoretical model for clinical practice. Frontiers in Psychology 12: 645246. 33796055 10.3389/fpsyg.2021.645246 PMC8008322

[bibr26-02698811221099650] gov.uk (2019) Crime Survey for England and Wales (CSEW) 2019. Available at: https://www.gov.uk/government/statistics/drug-misuse-findings-from-the-2018-to-2019-csew

[bibr27-02698811221099650] gov.uk (2010) Crime Survey for England and Wales (CSEW) 2010. Available at: https://www.gov.uk/government/statistics/drug-misuse-declared-findings-from-the-2009-10-british-crime-survey-england-and-wales

[bibr28-02698811221099650] Haden M Emerson B Tupper KW (2016) A public-health-based vision for the management and regulation of psychedelics. Journal of Psychoactive Drugs 48(4): 243–252. 27430375 10.1080/02791072.2016.1202459

[bibr29-02698811221099650] Haijen ECHM Kaelen M Roseman L , et al. (2018) Predicting responses to psychedelics: A prospective study. Frontiers in Pharmacology 9: 897. 30450045 10.3389/fphar.2018.00897 PMC6225734

[bibr30-02698811221099650] Horyniak D Dietze P Degenhardt L , et al. (2013) The relationship between age and risky injecting behaviours among a sample of Australian people who inject drugs. Drug and Alcohol Dependence 132(3): 541–546. 23664499 10.1016/j.drugalcdep.2013.03.021

[bibr31-02698811221099650] Hübner S Haijen E Kaelen M , et al. (2020) Turn on, tune in, and drop out: Identifying predictors of attrition in observational psychedelic research (Preprint). Journal of Medical Internet Research 23: e25973. 10.2196/25973 PMC8367105 34319246

[bibr32-02698811221099650] Johnson M Richards W Griffiths R (2008) Human hallucinogen research: Guidelines for safety. Journal of Psychopharmacology (Oxford, England) 22(6): 603–620. 10.1177/0269881108093587 PMC3056407 18593734

[bibr33-02698811221099650] King G Zeng L (2001) Logistic regression in rare events data. Political Analysis 9(2): 137–163.

[bibr34-02698811221099650] Kopra EI Ferris JA Winstock AR , et al. (2022) Adverse experiences resulting in emergency medical treatment seeking following the use of magic mushrooms. Journal of Psychopharmacology. Epub ahead of print 7 April. DOI: 10.1177/02698811221084063. PMC9353971 35388724

[bibr35-02698811221099650] Leonard JB Anderson B Klein-Schwartz W (2018) Does getting high hurt? Characterization of cases of LSD and psilocybin-containing mushroom exposures to national poison centers between 2000 and 2016. Journal of Psychopharmacology (Oxford, England) 32(12): 1286–1294. 10.1177/0269881118793086 30182795

[bibr36-02698811221099650] Liechti ME (2017) Modern clinical research on LSD. Neuropsychopharmacology: Official Publication of the American College of Neuropsychopharmacology 42(11): 2114–2127. 28447622 10.1038/npp.2017.86 PMC5603820

[bibr37-02698811221099650] Malcolm B Thomas K (2021) Serotonin toxicity of serotonergic psychedelics. Psychopharmacology. Epub ahead of print 12 July. DOI: 10.1007/s00213-021-05876-x. 34251464

[bibr38-02698811221099650] Martin CM (1970) Caring for the ‘bad trip’. A review of current status of LSD. Hawaii Medical Journal 29(7): 555–560. 5490958

[bibr39-02698811221099650] McCabe OL (1977) Psychedelic drug crises: Toxicity and therapeutics. Journal of Psychedelic Drugs 9(2): 107–121.

[bibr40-02698811221099650] McGlothlin WH Arnold DO (1971) LSD revisited: A ten-year follow-up of medical LSD use. Archives of General Psychiatry 24(1): 35–49. 5538851 10.1001/archpsyc.1971.01750070037005

[bibr41-02698811221099650] Nayak SM Gukasyan N Barrett FS , et al. (2021) Classic psychedelic coadministration with lithium. Pharmacopsychiatry 54(5): 240–245. 34348413 10.1055/a-1524-2794

[bibr42-02698811221099650] Nichols DE (2016) Psychedelics. Pharmacological Reviews 68(2): 264–355. 26841800 10.1124/pr.115.011478 PMC4813425

[bibr43-02698811221099650] Nichols DE Grob CS (2018) Is LSD toxic? Forensic Science International 284: 141–145. 29408722 10.1016/j.forsciint.2018.01.006

[bibr44-02698811221099650] Oram M (2014) Efficacy and enlightenment: LSD psychotherapy and the drug amendments of 1962. Journal of the History of Medicine and Allied Sciences 69(2): 221–250. 22898355 10.1093/jhmas/jrs050

[bibr45-02698811221099650] Petranker R Anderson T Maier L , et al. (2020) Microdosing psychedelics: Subjective benefits and challenges, substance testing behavior, and the relevance of intention. Journal of Psychopharmacology 36: 85–96. 33591231 10.1177/0269881120953994

[bibr46-02698811221099650] Rucker JJ Jelen LA Flynn S , et al. (2016) Psychedelics in the treatment of unipolar mood disorders: A systematic review. Journal of Psychopharmacology (Oxford, England) 30(12): 1220–1229. 10.1177/0269881116679368 27856684

[bibr47-02698811221099650] Russ SL Carhart-Harris RL Maruyama G , et al. (2019) States and traits related to the quality and consequences of psychedelic experiences. Psychology of Consciousness: Theory, Research, and Practice 6(1): 1.

[bibr48-02698811221099650] Schmid Y Enzler F Gasser P , et al. (2015) Acute effects of lysergic acid diethylamide in healthy subjects. Biological Psychiatry 78(8): 544–553. 25575620 10.1016/j.biopsych.2014.11.015

[bibr49-02698811221099650] Spear LP (2000) The adolescent brain and age-related behavioral manifestations. Neuroscience and Biobehavioral Reviews 24(4): 417–463. 10817843 10.1016/s0149-7634(00)00014-2

[bibr50-02698811221099650] Steinberg L Albert D Cauffman E , et al. (2008) Age differences in sensation seeking and impulsivity as indexed by behavior and self-report: Evidence for a dual systems model. Developmental Psychology 44(6): 1764–1778. 18999337 10.1037/a0012955

[bibr51-02698811221099650] Strassman RJ (1984) Adverse reactions to psychedelic drugs. A review of the literature. The Journal of Nervous and Mental Disease 172(10): 577–595. 6384428 10.1097/00005053-198410000-00001

[bibr52-02698811221099650] Studerus E Gamma A Kometer M , et al. (2012) Prediction of psilocybin response in healthy volunteers. PLoS ONE 7(2): e30800. 10.1371/journal.pone.0030800 PMC3281871 22363492

[bibr53-02698811221099650] Williams DR (2018) Stress and the mental health of populations of color: Advancing our understanding of race-related stressors. Journal of Health and Social Behavior 59(4): 466–485. 30484715 10.1177/0022146518814251 PMC6532404

[bibr54-02698811221099650] Williams LM Brown KJ Palmer D , et al. (2006) The mellow years?: Neural basis of improving emotional stability over age. Journal of Neuroscience 26(24): 6422–6430. 16775129 10.1523/JNEUROSCI.0022-06.2006 PMC6674038

[bibr55-02698811221099650] Winstock AR Barratt MJ (2013) The 12-month prevalence and nature of adverse experiences resulting in emergency medical presentations associated with the use of synthetic cannabinoid products. Human Psychopharmacology 28(4): 390–393. 23881887 10.1002/hup.2292

[bibr56-02698811221099650] Winstock AR Barratt MJ Maier LJ , et al. (2019) Global Drug Survey (GDS) 2019 Key Findings Report. London: Global Drug Survey.

[bibr57-02698811221099650] Winstock AR Maier LJ Zhuparris A , et al. (2021) Global Drug Survey (GDS) 2021 Key Findings Report. London: Global Drug Survey.

[bibr58-02698811221099650] World Health Organization (2017) Depression and Other Common Mental Disorders: Global Health Estimates (No. WHO/MSD/MER/2017.2). Geneva: World Health Organization.

[bibr59-02698811221099650] Yaden DB Yaden ME Griffiths RR (2020) Psychedelics in psychiatry – Keeping the renaissance from going off the rails. JAMA Psychiatry 78: 469–470. 10.1001/jamapsychiatry.2020.3672 PMC8102315 33263720

[bibr60-02698811221099650] Yockey RA Vidourek RA King KA (2020) Trends in LSD use among US adults: 2015–2018. Drug and Alcohol Dependence 212: 108071. 32450479 10.1016/j.drugalcdep.2020.108071

[bibr61-02698811221099650] Zawilska JB Kacela M Adamowicz P (2020). NBOMes – highly potent and toxic alternatives of LSD. Frontiers in Neuroscience 14; 78. 32174803 10.3389/fnins.2020.00078 PMC7054380

