# Supplementary material for: Adverse experiences resulting in emergency medical treatment seeking following the use of lysergic acid diethylamide (LSD)
Source: J Psychopharmacol. 2022 Jun 7;36(8):956–64. doi: 10.1177/02698811221099650 (PMC9353972; doi:10.1177/02698811221099650)
Supplement: sj-docx-1-jop-10.1177_02698811221099650 – Supplemental material for Adverse experiences resulting in emergency medical treatment seeking following the use of lysergic acid diethylamide (LSD) [file sj-docx-1-jop-10.1177_02698811221099650.docx]

**Supplementary Methods – Multiple Correspondence Analysis**

Multiple Correspondence Analysis (MCA) is an exploratory data analysis tool used to identify patterns and associations between multiple categorical variables. It is a form of dimension reduction method and often seen analogous to Principal Component Analysis (PCA) for quantitative data. As in PCA and Factor Analysis, the first dimension explains most variance in the dataset with each additional dimension explaining less. Most commonly 2- or 3-dimensional solution is used, as each added dimension becomes less important and complicates interpretation of the data.

MCA produces a scatter plot or a factor map to visualize relationships between variables. Most correlated variables – in essence, variables or options that have commonly been selected together – occur near each other; while uncorrelated variables are plotted far from each other. The most common responses are generally closest to the centre of the grid or the origin (0:0) and conversely less common responses are further away.


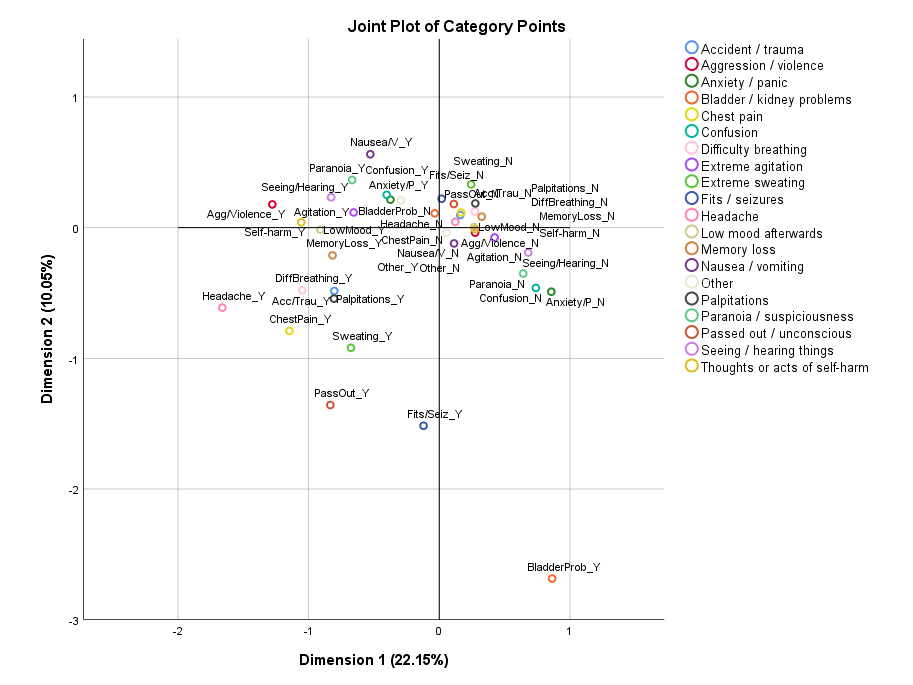
**Supplementary Figure S1. MCA Factor Map of Reported Symptoms**

3

2

1

A two-dimensional model explained a total of 32.2% of the variance (22.15% + 10.05%). “Yes” responses (indicated with a “Y”) are generally clustered in the left side of the y axis, and “No” responses (“N”) to the right. “Yes” responses are more spread out, reflecting that presenting a symptom was generally less common than not presenting one, as demonstrated by the median number of symptoms (5 vs. total options 20). Top left quadrant shows clusters of psychological and externalising symptoms as well as nausea. Specifically, aggression/violence, self-harm, and low mood afterwards were related (Cloud 1); as were anxiety/panic, confusion, nausea, and “Other” (Cloud 2). Encompassed by both clouds, seeing/hearing things, paranoia/suspiciousness, and extreme agitation, co-occurred with both of the two aforementioned set of symptoms. At the bottom left quadrant, Cloud 3 shows a cluster of more rare physiological symptoms as well as accident/trauma and memory loss.

**Supplementary Figure S2. MCA Factor Map of Reasons for Incidents**


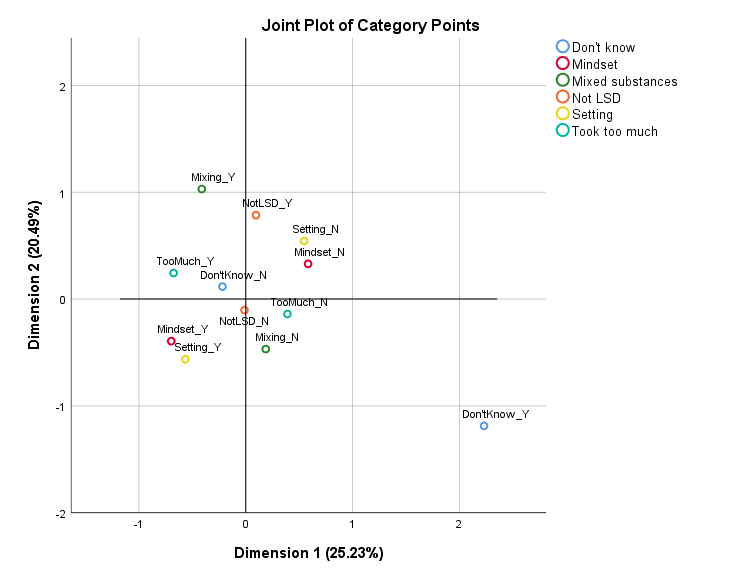


1

A two-dimensional model explained a total of 45.7% of the variance (25.23% + 20.49%). “Yes” responses generally clustered to the left side of y-axis; except for “Don’t’ know” on the far right, demonstrating selecting this option was expectedly linked to not ticking other reasons. Bottom left quadrant shows the two most reported reasons, wrong setting and wrong mindset, very commonly occurred together (Cloud 1).

**Supplementary Table S1.** Country of residence of past year LSD users

|  | **N** | **Valid %** |
| --- | --- | --- |
| Germany | 2,719 | 19.7 |
| United States | 2,703 | 19.6 |
| United Kingdom | 1,127 | 8.2 |
| Denmark | 1,039 | 7.5 |
| Canada | 793 | 5.8 |
| Australia | 693 | 5.0 |
| Brazil | 444 | 3.2 |
| Mexico | 417 | 3.0 |
| Switzerland | 394 | 2.9 |
| Colombia | 378 | 2.7 |
| Austria | 359 | 2.6 |
| Netherlands | 253 | 1.8 |
| New Zealand | 238 | 1.7 |
| Hungary | 177 | 1.3 |
| Norway | 173 | 1.3 |
| Italy | 159 | 1.2 |
| Poland | 134 | 1.0 |
| Scotland | 123 | 0.9 |
| Finland | 115 | 0.8 |
| Belgium | 103 | 0.7 |
| Greece | 102 | 0.7 |
| Sweden | 99 | 0.7 |
| Argentina | 92 | 0.7 |
| Ireland | 92 | 0.7 |
| France | 81 | 0.6 |
| Portugal | 79 | 0.6 |
| Russian Federation | 56 | 0.4 |
| Czech Republic | 55 | 0.4 |
| Spain | 52 | 0.4 |
| Estonia | 40 | 0.3 |
| Serbia and Montenegro | 38 | 0.3 |
| Wales | 38 | 0.3 |
| Croatia | 36 | 0.3 |
| Iceland | 22 | 0.2 |
| Other | 346 | 2.5 |
| Total | 13,769 | 100.0 |

**Supplementary Table S2.** Country of residence of EMT seekers

|  | **N** | **Valid %** |
| --- | --- | --- |
| Germany | 19 | 18.6 |
| United States | 19 | 18.6 |
| United Kingdom | 12 | 11.8 |
| Denmark | 9 | 8.8 |
| Australia | 8 | 7.8 |
| Brazil | 6 | 5.9 |
| Austria | 5 | 4.9 |
| Canada | 3 | 2.9 |
| France | 3 | 2.9 |
| Colombia | 2 | 2.0 |
| Belgium | 1 | 1.0 |
| Ecuador | 1 | 1.0 |
| Finland | 1 | 1.0 |
| Guatemala | 1 | 1.0 |
| Israel | 1 | 1.0 |
| Italy | 1 | 1.0 |
| New Zealand | 1 | 1.0. |
| Norway | 1 | 1.0 |
| Poland | 1 | 1.0 |
| Russian Federation | 1 | 1.0 |
| Scotland | 1 | 1.0 |
| Slovakia | 1 | 1.0 |
| Sweden | 1 | 1.0 |
| Switzerland | 1 | 1.0 |
| Ukraine | 1 | 1.0 |
| Wales | 1 | 1.0 |
| Total | 102 | 100 |
